# Calculating shear viscosity with confined non-equilibrium molecular dynamics: a case study on hematite – PAO-2 lubricant†

**DOI:** 10.1039/d3ra06929j

**Published:** 2023-11-21

**Authors:** Dimitrios Mathas, Davide Sarpa, Walter Holweger, Marcus Wolf, Christof Bohnert, Vasilios Bakolas, Joanna Procelewska, Joerg Franke, Philipp Rödel, Chris-Kriton Skylaris

**Affiliations:** a Department of Chemistry, University of Southampton Highfield Southampton SO17 1BJ UK C.Skylaris@soton.ac.uk; b Mechanical Engineering Department, University of Southampton Highfield Southampton SO17 1BJ UK; c Schaeffler Technologies AG & Co. KG Herzogenaurach Germany; d Department of Mechanical and Process Engineering, RPTU Kaiserslautern-Landau Gottlieb-Daimler-Str. 67663 Kaiserslautern Germany

## Abstract

The behaviour of confined lubricants at the atomic scale as affected by the interactions at the surface–lubricant interface is relevant in a range of technological applications in areas such as the automotive industry. In this paper, by performing fully atomistic molecular dynamics, we investigate the regime where the viscosity starts to deviate from the bulk behaviour, a topic of great practical and scientific relevance. The simulations consist of setting up a shear flow by confining the lubricant between iron oxide surfaces. By using confined Non-Equilibrium Molecular Dynamics (NEMD) simulations at a pressure range of 0.1–1.0 GPa at 100 °C, we demonstrate that the film thickness of the fluid affects the behaviour of viscosity. We find that by increasing the number of lubricant molecules, we approach the viscosity value of the bulk fluid derived from previously published NEMD simulations for the same system. These changes in viscosity occurred at film thicknesses ranging from 10.12 to 55.93 Å. The viscosity deviations at different pressures between the system with the greatest number of lubricant molecules and the bulk simulations varied from −16% to 41%. The choice of the utilized force field for treating the atomic interactions was also investigated.

## Abstract 2

Atomistic simulations show for the first time how the variation of a lubricant's film thickness affects viscosity and at what point it eventually becomes comparable with the bulk simulation of a lubricant, at different pressures and shear regimes.

## Graphical abstract

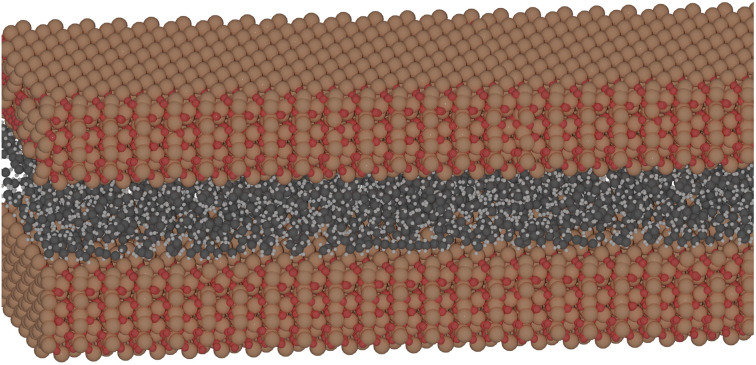

## 1 Introduction

The rheological properties of lubricants play a crucial role in their applicability to a wide range of systems from the automotive industry to materials development.^1–3^ Lubricants are employed for antiwear, as rust and corrosion inhibitors, as brake fluids and as friction modifiers. Within all of these applications, the most fundamental property is viscosity, which allows the formation of a film between two surfaces; it is defined as the loss of linear momentum in the direction perpendicular to the flow. Understanding how chemistry affects viscosity is an active field of research and has been investigated *via* experiments and more recently, *via* computer simulations. The main methods to computationally study rheological properties include the equilibrium molecular dynamics coupled with the Green–Kubo equations^4,5^ (EMD-GK), where viscosity is expressed in terms of integrals of the autocorrelation function of the pressure tensor, and the non-equilibrium molecular dynamics (NEMD), where viscosity is computed by imposing a shear field. In these methods, viscosity is calculated using a bulk liquid model. For a detailed description of these methods see ref. 6 and 7, respectively. Although these methods work very well in many cases for a variety of different lubricants and conditions,^8–14^ it is desirable to have a more realistic model where the liquid of interest is confined between explicitly defined walls which aim to resemble as closely as possible real-life applications under different operational conditions. This method is known as confined NEMD simulation and has been applied to various systems including Lennard-Jones particles,^15^ linear and branched hydrocarbon oils,^16–18^ esters,^19^ acids^20^ and many more.^21–27^ Within the formulation of confined NEMD, there are several ways to derive the shear stress which is essential for calculating viscosity. There exist several methods to do so including the Irving–Kirkwood method (IK),^28^ the method of planes (MoP),^29^ the average shear force over area^30^ and the direct shear stress from average atom shear stress of lubricant atoms. In this work, we employed the direct shear method, due to its conceptual simplicity, as implemented in the LAMMPS^31^ software for calculating viscosity at various operational conditions. We investigated varying pressure, the applied shear rate and the film thickness of the confined lubricant. This method calculates shear viscosity as:1
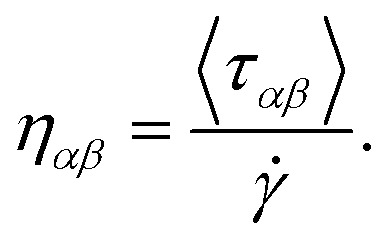
where *η*_*αβ*_ is the viscosity, 〈*τ*_*αβ*_〉 is the shear stress that is averaged over the ensemble, *αβ* ≡ *x*, *y*, *z* and *γ̇* is the applied shear rate. In such context, the simulation box is periodic in the *x* and *y*-dimension and non-periodic in the *z*-dimension. In our group, we have previously studied a bulk liquid made of 9,10-dimethyloctadecane at different operational conditions employing both EMD and NEMD simulations.^32^ We are now moving towards a more realistic system which includes 9,10-dimethyloctadecane molecules confined between two α-Fe_2_O_3_ (hematite) (001) slabs. We study the effect of three different pressures (0.1, 0.5 and 1 GPa), two shear rates (10^7.5^ and 10^8.5^ s^−1^) and the predictive power of two force fields L-OPLS-AA and ReaxFF, the latter being a reactive force field. We present the advantages and discuss the limitations of this method. We then compare these results against bulk simulations on the same liquid published in our previous study,^32^ where bulk simulations were compared to experimental measurements of viscosity at pressures up to 1 GPa. Our goal is to study the impact of film thickness, shear rate and force field on the viscosity of confined lubricant compared to the bulk system.

## 2 Results

### 2.1 Compression stage

**Fig. 1:**
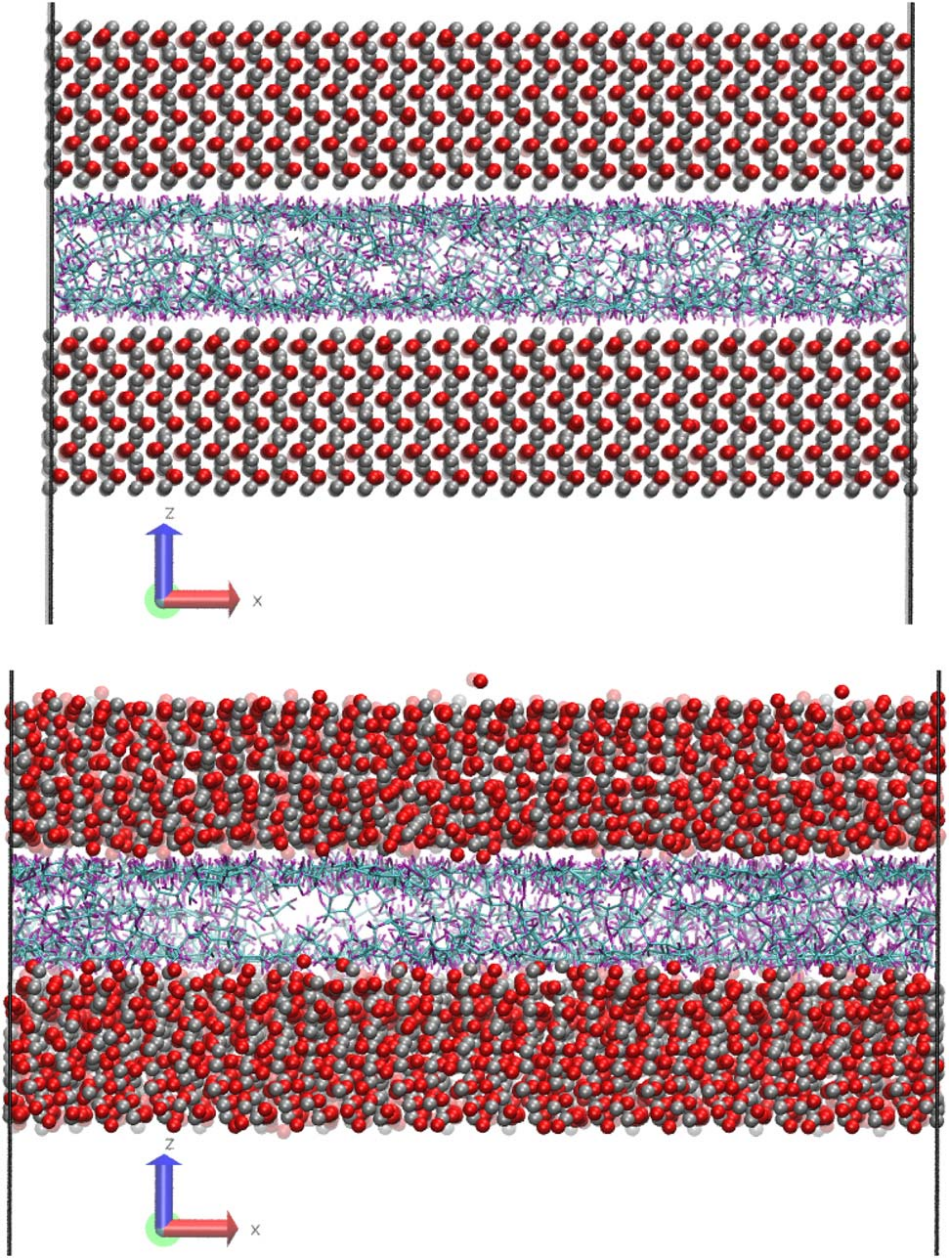
(top) Molecular snapshot of system 1 (100 lubricant molecules), with L-OPLS-AA at 0.1 GPa and 100 °C after the compression stage. (bottom) Molecular snapshot of system 1 (100 lubricant molecules), with ReaxFF at 0.1 GPa and 100 °C after the compression stage. Carbon atoms are coloured cyan, hydrogen atoms with purple, oxygen atoms with red and iron atoms with silver.

Fig. 1 shows a molecular snapshot of the systems studied after successful compression at a pressure of 0.1 GPa at 100 °C by using the L-OPLS-AA force field. As can be seen from the illustrations, the atomic arrangement of the iron oxide surface is well-preserved as their bonds are described by a bonded force field. On the other hand, at the ReaxFF level at a pressure of 0.1 GPa at 100 °C, the iron oxide atoms have more freedom to move as there are no explicit bonds between atoms. Interestingly, for both force fields, we can see the formation of a monolayer of lubricant molecules near the lubricant–surface interface, which has also been observed in other studies using different systems of surface and lubricants.^23,30^

### 2.2 Density profiles

**Fig. 2:**
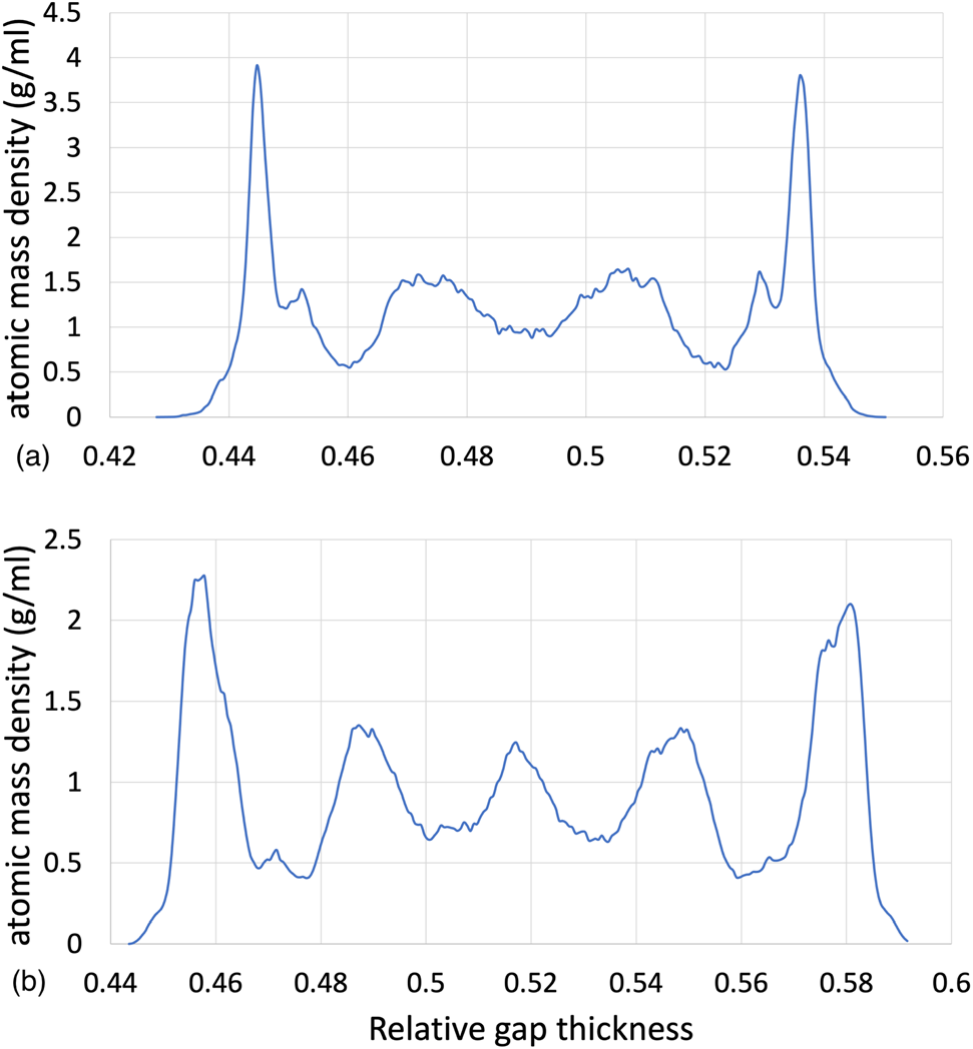
Atomic mass density profile of system 2 (200 lubricant molecules) at 100 °C, a log *γ̇* of 8.50 and a pressure of 0.5 GPa with (a) ReaxFF and (b) L-OPLS-AA.

**Fig. 3:**
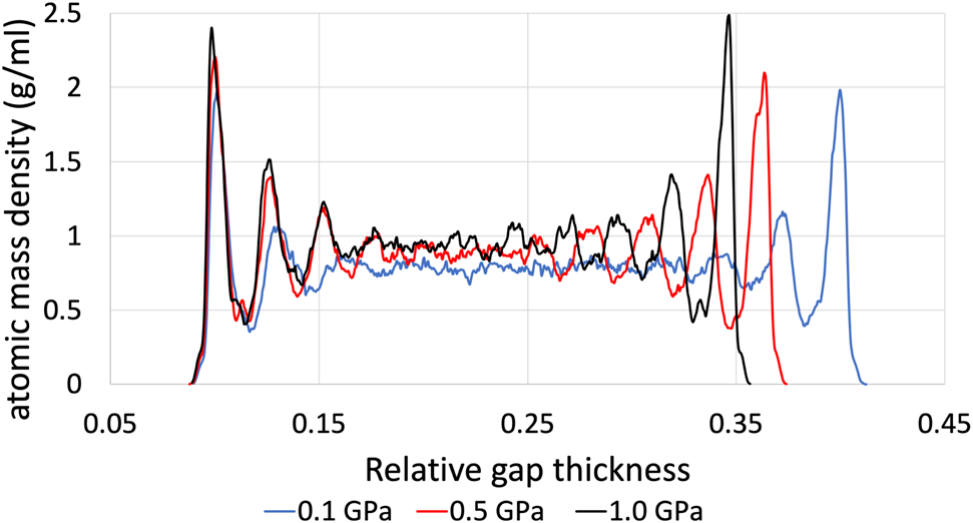
Atomic mass density profile of system 3 (450 lubricant molecules) with L-OPLS-AA at 100 °C, a log *γ̇* of 8.50 and a pressure range of 0.1 GPa, 0.5 GPa and 1.0 GPa.

The structural analysis of the layering of the fluid due to the presence of hematite slabs, seen in the confined simulations is presented in the following figures, quantified as density profiles. The density profiles were acquired by averaging over the last 5000 iterations (1.25 ps for ReaxFF and 5 ps for L-OPLS-AA) during the shearing production run. Fig. 2 shows the atomic mass density profiles in the *z*-direction, for system 2 (200 lubricant molecules) at 0.5 GPa and a log *γ̇* of 8.50, by using the ReaxFF and L-OPLS-AA force fields. The term “relative gap thickness” is a dimensionless parameter that quantifies the normalized bin sizes ranging from 0 to 1, which are employed to partition and subsequently calculate density along the *z*-direction. When multiplied by the box dimension, it yields the distance in Angstrom. The oscillatory atomic mass density profile closer to the surface indicates stronger layering of the lubricant when compared to the centre of the film. These oscillations are similar to those from confined NEMD simulations of squalane.^23^ Additionally, by looking at the density profile closer to the surface, stronger layering was observed in ReaxFF than L-OPLS-AA for the same conditions that were tested. Then, Fig. 3 shows the atomic mass density profile in the *z*-direction, for system 3 (450 lubricant molecules) at a range of pressures (0.1 to 1.0 GPa) and a log *γ̇* of 8.50, by using the L-OPLS-AA force field. By comparing these density profiles, the following can be said. Firstly, by increasing the applied pressure, density oscillations become more apparent, and as expected, the overall densities increase as well. Secondly, the increase in pressure shrinks the total density profile which is equivalent to the volume contraction where the fluid is confined. Finally, the average density of the confined fluid (blue line in Fig. 3) is in excellent agreement with the respective density of the bulk liquid simulations at 0.1 GPa, with both densities being equal to 0.79 g ml^−1^.

Moreover, the average densities of the three systems (L-OPLS-AA case) with 100, 200 and 450 lubricant molecules at 0.5 GPa and a shear rate of 10^8.5^ s^−1^ were found to be equal to 0.90 ± 0.52, 0.90 ± 0.50 and 0.89 ± 0.35 g ml^−1^, respectively. By comparing the standard deviation of these average densities for the three systems, we see that by increasing the number of lubricant molecules, the density oscillations decrease.

### 2.3 Film thicknesses

Table 1 shows the different average film thicknesses, which were calculated before the shearing stage, with their respective standard deviation. We see that by increasing the number of confined lubricant molecules within the two iron oxide surfaces, the relative standard deviation (coefficient of variation, *i.e.*, standard deviation divided by the mean thickness) decreases. For example, for the case of L-OPLS-AA force field at 0.1 GPa, the relative standard deviations of systems 1, 2 and 3 are 0.6%, 0.5% and 0.4% respectively.

**Table 1:** Average film thickness L-OPLS-AA (last 2 ns) and ReaxFF (last 0.5 ns) simulations

Lubricant molecules	Film thickness (Å)	Force field	*P* (GPa)
100	13.70 ± 0.08	L-OPLS-AA	0.1
200	25.40 ± 0.12	L-OPLS-AA	0.1
450	55.93 ± 0.22	L-OPLS-AA	0.1
100	12.45 ± 0.04	ReaxFF	0.1
200	19.59 ± 0.06	ReaxFF	0.1
100	12.27 ± 0.04	L-OPLS-AA	0.5
200	22.75 ± 0.06	L-OPLS-AA	0.5
450	49.54 ± 0.09	L-OPLS-AA	0.5
100	11.06 ± 0.03	ReaxFF	0.5
200	17.91 ± 0.03	ReaxFF	0.5
100	11.44 ± 0.04	L-OPLS-AA	1.0
200	21.50 ± 0.05	L-OPLS-AA	1.0
450	46.43 ± 0.07	L-OPLS-AA	1.0
100	10.12 ± 0.02	ReaxFF	1.0
200	17.35 ± 0.03	ReaxFF	1.0

We can also see that by applying higher pressure loads at the upper outermost layer of the iron oxide slab, for a given system that has the same number of lubricant molecules and regardless of the force field used, the standard deviation of the average film thickness decreases. This can be explained by the fact that the increase in pressure leads to less freedom of movement for the lubricant molecules while overcoming repulsion forces between the lubricant and the walls.

Compared to L-OPLS-AA, ReaxFF simulations resulted in a thinner film for the same systems and conditions. This can be explained by the fact that ReaxFF allows atoms to come closer into contact with the surface (see Fig. 1a and b). It appears that this difference is more evident at higher pressures (Table 1). It is also important to notice that the hematite density is better described by ReaxFF compared to the L-J potential. This is linked to the better description of the forces between atoms in the slabs in the ReaxFF compared to the simple L-J potential.

**Fig. 4:**
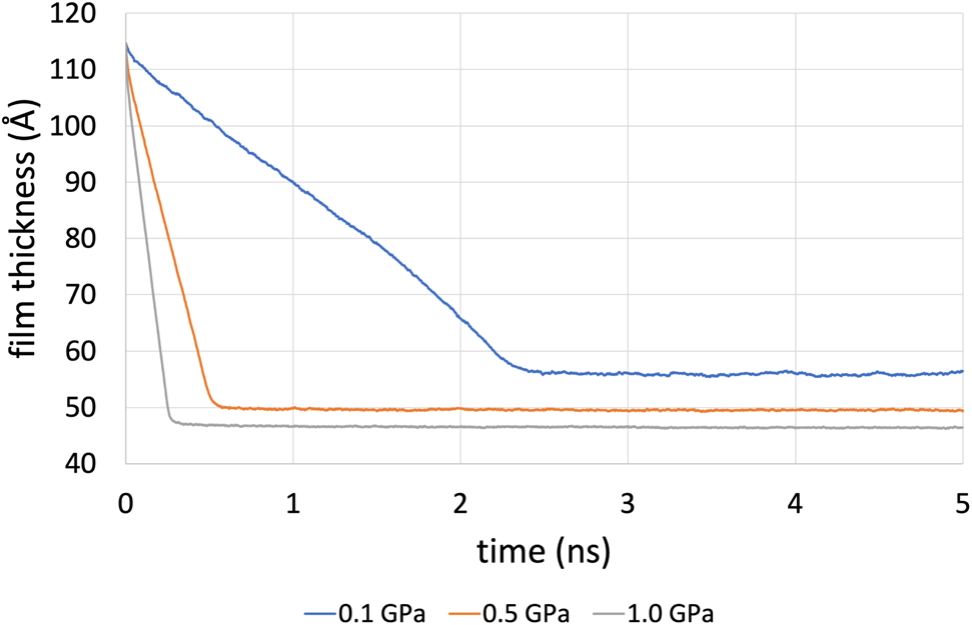
Film thickness of system 3 (450 lubricant molecules), with L-OPLS-AA at 100 °C during the compression stage of 5 ns. For this case, compression is slower compared to systems 1 and 2, as there are more confined lubricant molecules between the iron oxide slabs. This results in increased repulsion forces arising from the lubricant.

Fig. 4 shows the compression stage during the time evolution of the simulation when using the L-OPLS-AA force field for system 3 (450 lubricant molecules). It was found that the time required to reach a fully compressed state increases with the system's size (see, ESI†). This can be explained by the increase of repulsion forces arising from the lubricant. Increasing the pressure from 0.1 GPa to 1.0 GPa decreases the compression time from 3 ns to 1 ns.

It was also observed that the ReaxFF force field is slower than the L-OPLS-AA force field in terms of time required for reaching equilibrium during compression and overall time performance (approximately 44 times slower than L-OPLS-AA per femtosecond).

### 2.4 Statistical analysis of viscosity results

Tables 2 and 3 show the viscosity results obtained from molecular simulation with the L-OPLS-AA and ReaxFF force field at 100 °C, respectively.

**Table 2:** Viscosity results of 9,10-dimethyloctadecane at 100 °C using L-OPLS-AA and comparison with bulk simulations.^32^ Note that the deviation is with respect to the bulk value at the same operational conditions of temperature, pressure and shear rate. Pressure values are in GPa, viscosity in mPa s

Molecules	log(*γ̇*)	*P*	Viscosity	Deviation, %	Bulk viscosity
100	7.50	0.1	27.8	300	6.94
100	7.50	0.5	664	425	126
100	7.50	1.0	1.95 × 10^3^	48	1.32 × 10^3^
100	8.50	0.1	15.0	132	6.47
100	8.50	0.5	117	50	78.1
100	8.50	1.0	236	−13	272
200	7.50	0.1	22.3	221	6.94
200	7.50	0.5	431	241	126
200	7.50	1.0	1.56 × 10^3^	19	1.32 × 10^3^
200	8.50	0.1	11.4	76	6.47
200	8.50	0.5	103	33	78.1
200	8.50	1.0	223	−18	272
450	7.50	0.1	6.50	−6	6.94
450	7.50	0.5	178	41	126
450	7.50	1.0	1.31 × 10^3^	−0.4	1.32 × 10^3^
450	8.50	0.1	6.21	−4	6.47
450	8.50	0.5	85.9	10	78.1
450	8.50	1.0	230	−16	272

**Table 3:** Viscosity results of 9,10-dimethyloctadecane at 100 °C using ReaxFF at a pressure range of 0.1 to 1.0 GPa and a log(*γ̇* [s^−1^]) of 8.5. Pressure values are in GPa, viscosity in mPa s

Molecules	System	log(*γ̇*)	*P*	Viscosity
100	1	8.50	0.1	294
100	1	8.50	0.5	540
100	1	8.50	1.0	764
200	2	8.50	0.1	181
200	2	8.50	0.5	335
200	2	8.50	1.0	607

When we take into consideration the effect on viscosity by varying the number of confined lubricant molecules within the surfaces, it can be seen that in both force field cases, when applying the same pressure and shear rate, viscosity decreases when the number of lubricant molecules increases. This is due to an increased film thickness which allows the molecule to have more freedom but also reduces the surface interactions. When we refer to “surface interactions”, we are specifically addressing the non-bonded interactions (Lennard-Jones and electrostatic) between the surface and the lubricant molecules.

Increasing the shear rate resulted in almost all cases, in shear thinning. The only exception was the case of system 3 (L-OPLS-AA), where at a pressure of 0.1 GPa, the viscosity values for both applied shear rates were very close, as for this case, simulations were very close to the Newtonian regime, where viscosity does not depend on the applied shear rate and has a constant value.

We also studied the effect of pressure on viscosity, which increased when a higher pressure was applied externally. The ReaxFF force field predicts a higher viscosity compared to the L-OPLS-AA force field but we observe the same change in viscosity behaviour qualitatively. The deviation between the two force fields is more apparent in lower pressures. For example, at *P* = 0.5 GPa and *γ̇* = 108.5 s^−1^ (system 2) ReaxFF overestimated viscosity by 224% compared to L-OPLS-AA, while at *P* = 1.0 GPa and *γ̇* = 108.5 s^−1^ (system 2) ReaxFF overestimated viscosity by 172%.

We also see that by increasing the number of molecules the viscosity approaches the bulk value reported in a previous study.^32^ For those interested, the overestimation of viscosity compared to the experiments and the comparison with experiments regarding the Newtonian limit are discussed in ref. 32.

**Fig. 5:**
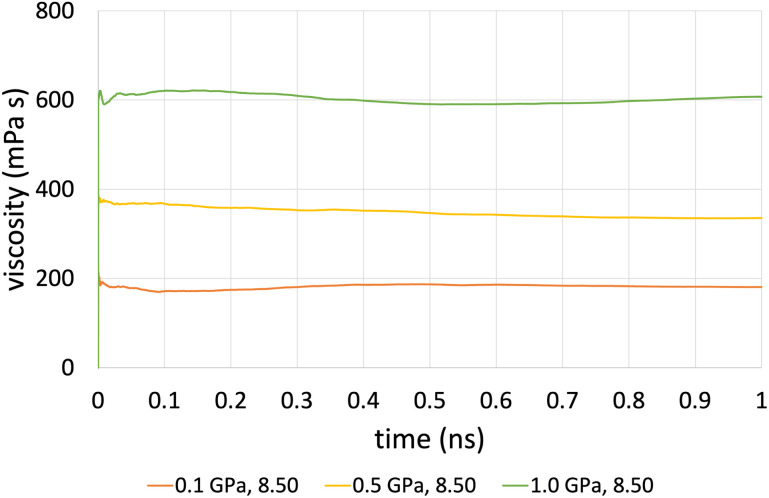
Average viscosity of system 2 (200 lubricant molecules), with ReaxFF at a pressure range from 0.1 to 1.0 GPa at 100 °C and at a shear rate of 10^8.5^ s^−1^.

The time-averaged viscosity during the shearing stage of the production run for system 3 can be seen in Fig. 5 for the case of ReaxFF. The overall trend was that simulations at a shear rate of 10^7.5^ s^−1^ did not converge, compared to those at a shear rate of 10^8.5^ s^−1^ (orange, yellow and green lines, respectively), which converged after 1 ns. This is due to the fact that at lower shear rates, the equilibrium fluctuations become comparable with the non-equilibrium response, which results in a lower signal-to-noise ratio, and as a result, we need to increase the total simulation time and the sampling interval of viscosity, which in the end is the whole duration of the simulation.

**Fig. 6:**
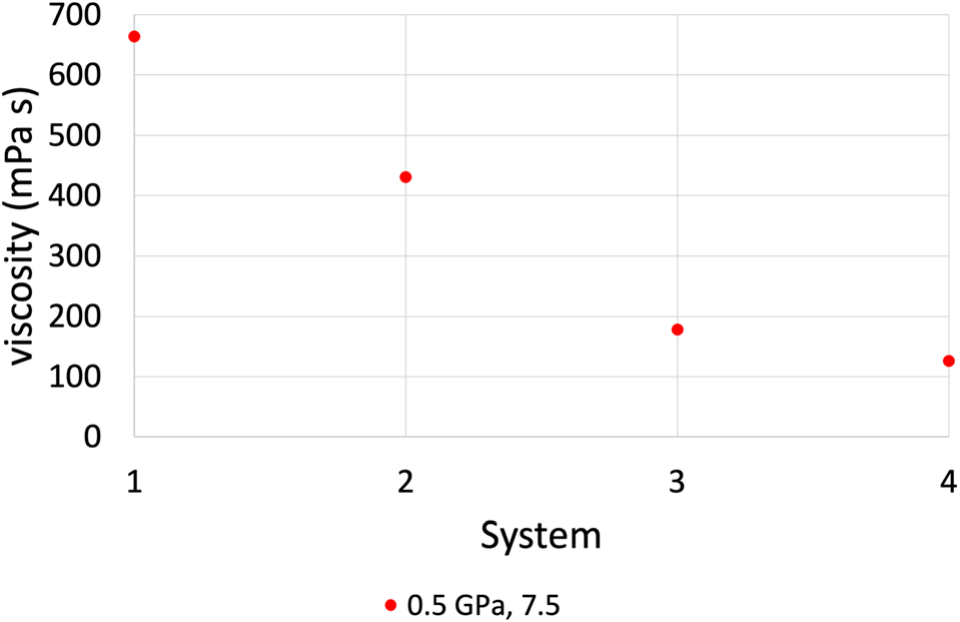
Viscosity results comparison between confined NEMD simulations (system 1, 2 and 3) and bulk NEMD simulations (system 4) at *P* = 0.5 GPa and log(*γ̇* [s^−1^]) = 7.5, by using the L-OPLS-AA force field. As we increase the number of confined lubricant molecules we approach bulk behaviour of viscosity. Systems 1, 2 and 3 contain 100, 200 and 450 lubricant molecules, respectively.

Fig. 6 shows the behaviour of viscosity as a function of the number of lubricant molecules confined within the iron oxide surfaces, which are compared against the bulk NEMD simulations (system 4). The overall trend of viscosity, as we reach a film thickness close to the bulk simulation, is that we approach the bulk values of viscosity. This means that if the number of lubricant molecules is sufficient, confined NEMD can also give reliable viscosity results that are close to the bulk viscosity values. The only exception was the case of the highest pressure of 1.0 GPa at a high shear rate of 10^8.5^ s^−1^, where all systems gave very similar results, this indicates that confinement effects are less prominent at sufficiently high shear rates that depend on the applied pressure.

In principle one should increase the number of molecules further to assess possible oscillatory behaviour of the convergence but this is at the moment out of our computational capabilities. Nonetheless, it's worth noting that our analysis indicates a monotonic trend rather than any oscillatory behaviour in the viscosity as the number of molecules increases. While we are unable to explore larger systems due to their computational cost, the available data does not suggest any oscillatory nature in the convergence of viscosity values.

It is also important to notice that the viscosity exhibits a significant variation, changing by more than sixfold, from 1320 to 270 mPa s, when the strain rate is increased tenfold, shifting from 10^7.5^ to 10^8.5^ s^−1^ at 1 GPa. Interestingly, when the number of molecules is altered, moving from 100 to 450 while keeping the strain rate constant at 10^7.5^ s^−1^ and at 1 GPa, the change in viscosity is comparatively modest, being less than a twofold difference, shifting from 1950 to 1310 mPa s. Furthermore, we analyzed the effect of pressure on the bulk viscosity. Notably, a tenfold change in pressure, from 0.1 to 1 GPa, led to a substantial alteration in viscosity. At a strain rate of 10^8.5^ s^−1^, the change amounted to approximately 40-fold, while at 10^7.5^ s^−1^, it exceeded 200-fold. In contrast, when modulating confinement and the associated shift in film thickness within the range of approximately 10–50 angstroms, we observed a notably weaker influence on viscosity compared to similar adjustments in other parameters, such as strain rate and pressure.

Comparing viscosity values in Tables 3 and 2 we observe the same behaviour with L-OPLS-AA force field qualitatively, as viscosity decreased when the number of lubricant molecules increased. L-OPLS-AA predicts viscosity values to be closer to experiments than ReaxxFF, although the latter could be used for studying possible chemical reactions at high-pressure-temperature regimes. ESI figures related to the film thickness, viscosity and velocity profiles of the different systems in this study are available in the ESI.†

### 2.5 Radius of gyration and gyration moments

To gain deeper insights into the intramolecular orientation, we analysed the behaviour of lubricant molecules. Specifically, we computed the average radius of gyration (*R*_g_) over the last nanosecond (ns) of the simulations (Table 4).

**Table 4:** Radius of gyration and gyration moments for L-OPLS-AA simulation at 0.1 GPa and at shear rate of 10^8.5^ s^−1^

Simulation	*R*_g_	*λ*_*x*_	*λ*_*y*_	*λ*_*z*_
L-OPLS-AA 100	28.9	11.0	355.3	471.1
L-OPLS-AA 200	29.6	48.0	356.6	474.4
L-OPLS-AA 450	32.8	245.3	357.0	475.0

As anticipated, we observed that *R*_g_ increased as the number of lubricant molecules, and consequently the film thickness, increased. This behaviour aligns with our expectations as the confinement is less strict when the number of lubricant molecules increases.

However, what truly piqued our interest was the significant role played by the *λ*_*x*_ component of the gyration tensor, which corresponds to the component aligned with the flow direction. Our analysis revealed that as the film thickness increased, this component made the greatest contribution to the change in *R*_g_. In practical terms, this implies that as the lubricant film becomes thicker, the molecules within it exhibit a pronounced elongation along the direction of the flow. When we compare this observation with the viscosity values, it becomes apparent that as molecules become more elongated in the direction of the flow, their viscosity tends to decrease.

## 3 Discussion

In this study, we have performed confined non-equilibrium molecular dynamics with two force fields (L-OPLS-AA and ReaxFF) to understand the microscopic behaviour and shear viscosity of 9,10-dimethyloctadecane molecules confined between two hematite slabs at various pressures, shear rates and film thicknesses. L-OPLS-AA is a well-established force field known for its accuracy in viscosity simulations for various classes of lubricants. However, it lacks parameters for surfaces, which are crucial for simulating industrial applications involving lubricants. In many cases, in confined L-OPLS-AA simulations surfaces are fixed and the interactions with the lubricant employ a simple Lennard-Jones (L-J) approach. ReaxFF, on the other hand, offers a unique advantage in that it treats both hematite surfaces and lubricants on equal footing. This allows it to capture more physics and chemistry at the interface, making it suitable for simulating systems with complex surface–lubricant interactions. Given its reactivity, ReaxFF has the potential to provide insights into chemical aspects of lubricant behaviour, which is essential in certain scenarios. We have shown that the film thickness affects viscosity and as we increase the number of lubricant molecules, we approach the viscosity value of the bulk fluid, that was obtained with NEMD. In particular, viscosity values were in good agreement between the two methods (NEMD and confined NEMD), when there were enough lubricant molecules confined within the walls. For example, at a shear rate of 10^7.5^ s^−1^ and 0.1 GPa at a film thickness of 25 Å the simulation already deviates from the bulk behaviour by 221%. The only exception that was observed was in the high pressure (1.0 GPa)–high shear rate (10^8.5^ s^−1^) regime, where the different film thicknesses had no influence on viscosity. This indicates confinement effects are less prominent at sufficiently high shear rates that depend on the applied pressure. The density profiles in the *z*-direction have been investigated. For both force fields we observed the formation of a monolayer of lubricant molecules near the lubricant–surface interface. We also observed a reduction in the oscillation of the density when increasing the number of molecules. The liquid densities calculated for both force fields are in agreement with experimental data but ReaxFF predicts hematite density better than the simple L-J which is employed to describe the hematite in the case of L-OPLS-AA. We also found that the ReaxFF force field overestimates viscosity when compared to the L-OPLS-AA force field but the same change in viscosity behaviour with simulation parameters is observed qualitatively. L-OPLS-AA appears to have a better prediction of viscosities of organic lubricants under confinement compared to ReaxFF, probably due to being parameterised specifically for these types of molecules. The deviation between the two force fields was more apparent at lower pressures. For example, at *P* = 0.5 GPa and *γ̇* = 108.5 s^−1^ (system 2–200 lubricant molecules) ReaxFF overestimated viscosity by 224% compared to L-OPLS-AA, while at *P* = 1.0 GPa and *γ̇* = 108.5 s^−1^ (system 2–200 lubricant molecules) ReaxFF overestimated viscosity by 172%. Our findings suggest that capturing more physics and chemistry, as offered by ReaxFF, does not necessarily translate into better accuracy in describing viscosity. This phenomenon bears a resemblance to findings in biomolecular simulations, exemplified in the work of Bradshaw *et al.*^33^ In their study, they compared the AMOEBA force field with the GAFF force field to investigate the hydration free energy of various neutral organic compounds. Interestingly, their research revealed that despite AMOEBA's departure from the pairwise additive models of electrostatics, it was only able to achieve free energy values on par with the traditional GAFF. This achievement, however, required extensive and meticulous parameter optimization. This observation raises important questions about the parametrization of force fields and the balance between complexity and simplicity. The ReaxFF force field, in our case, exhibits a notably higher level of complexity when juxtaposed with classical force fields like L-OPLS-AA. Consequently, this heightened complexity translates into a more intricate and time-consuming parameterisation process. As the field of molecular simulations evolves, the question of how to improve force field accuracy and applicability remains. Machine learning (ML) force fields are emerging as a promising avenue, and it is indeed worth considering their potential for addressing some of the limitations associated with traditional force fields.

Our simulations provide new insights into rheological properties that can occur in concentrated contacts but are difficult to study experimentally. The simulation protocols and workflows we have developed in this work can be used to improve the understanding of lubricant behaviour at the atomic scale, providing insights into the fundamental mechanism of very thin lubricant films.

## 4 Methods

Hematite (α-Fe_2_O_3_) is a well-known material and represents a model for surfaces present in rolling bearings and gears. It is the most stable and common form of iron oxide under ambient conditions, and its (001) surface is the most stable surface according to DFT calculations.^34–36^ In addition, iron oxide is known to form in tribological systems of steel under various conditions. For this reason, it is chosen as a surface for our system. We have chosen 9,10-dimethyloctadecane as model liquid due to being the main component of the industrially relevant PAO-2 lubricant. Three different systems were generated consisting of either 100, 200, or 450 9,10-dimethyloctadecane molecules between two slabs of iron oxide (2700 molecules of α-Fe_2_O_3_ in total). The numbers of molecules were chosen such as they would model film thicknesses found in experiments (200 molecules) in bulk (450 molecules) and an extreme condition which is hard to obtain experimentally (100 molecules). The three molecular systems were generated by using an in-house modified version of LAMMPS_builder.^37,38^

### 4.1 Simulation details

The three systems were studied using the open-source LAMMPS software^31^*via* two different force fields L-OPLS-AA^39^ and ReaxFF,^40,41^ parameters for ReaxFF were taken from ref. 42. The L-OPLS-AA force field, which is among the most popular force fields for liquid simulations, is a bonded all-atom force field. ReaxFF force field does not consider bonds explicitly as in L-OPLS-AA force field, but instead employs a bond-order formalism in conjunction with polarisable charge descriptions to describe both reactive and non-reactive interactions between atoms. This allows ReaxFF to accurately model both covalent and electrostatic interactions for a diverse range of materials. As a result, the ReaxFF force field can be used to study chemical reactions, as bonds can form and break during a simulation, something that cannot happen in bonded force fields, for example, during simulations with L-OPLS-AA.

The workflow included three distinct steps: an equilibration (reorientation) step, followed by compression and at last a shearing step. The shearing part consists of a steady state part followed by a production run.

### 4.2 L-OPLS-AA

The interactions within the fluid were modelled *via* L-OPLS-AA,^39^ while interactions between the iron oxide and fluid atoms were governed by Lennard-Jones (L-J) and electrostatic interactions (E.I). The E.I. and L-J parameters were those developed by Savio *et al.*^43^ and Berro *et al.*^44^ The α-Fe_2_O_3_ slabs were restrained in their crystal structure using harmonic restrains with a spring constant of 130 kcal mol^−1^.^44^ A 1 fs timestep was used during the entire workflow.

The equilibration and molecular reorientation step was achieved by an energy relaxation process, followed by a run of 8 ns in the canonical (NVT) ensemble that included a Langevin thermostat,^45^ which was applied to the lubricant atoms, to control the temperature at 373 K with a time constant of 0.1 ps. To allow molecular reorientation of the lubricant, the outermost layer of iron atoms of the upper and lower iron oxide slabs was kept frozen for the whole duration of the simulation.

Three independent trajectories were produced by randomizing the positions and velocities of the initial configuration. This was achieved by heating and then cooling the configuration through separate cycles. These heat-quench cycles^46^ for simulations at 373 K were performed from 373 K to *T* = 375, 380 and 385 K, respectively, during 1 ns runs in the NVT ensemble, after which the systems were immediately quenched back to 373 K during another 1 ns run in the NVT ensemble.

The second step of the simulation included the application of external pressure (0.1, 0.5 and 1.0 GPa) at the outermost layer of iron atoms of the upper slab in order to compress the systems. The pressure values were chosen to be in the range of the applied pressure in bulk NEMD simulations that were used in a previous study for the same liquid.^32^ This was achieved during 5–8 ns runs where the outermost bottom layer of the lower iron oxide slab was kept frozen for the whole duration of the simulation. The simulation ran for long enough until the film thickness reached a negligible fluctuation (≤0.05 Å). Then, the film thickness values during the last 2 ns were used to determine the average film thickness value needed for the next step of shearing.

**Fig. 7:**
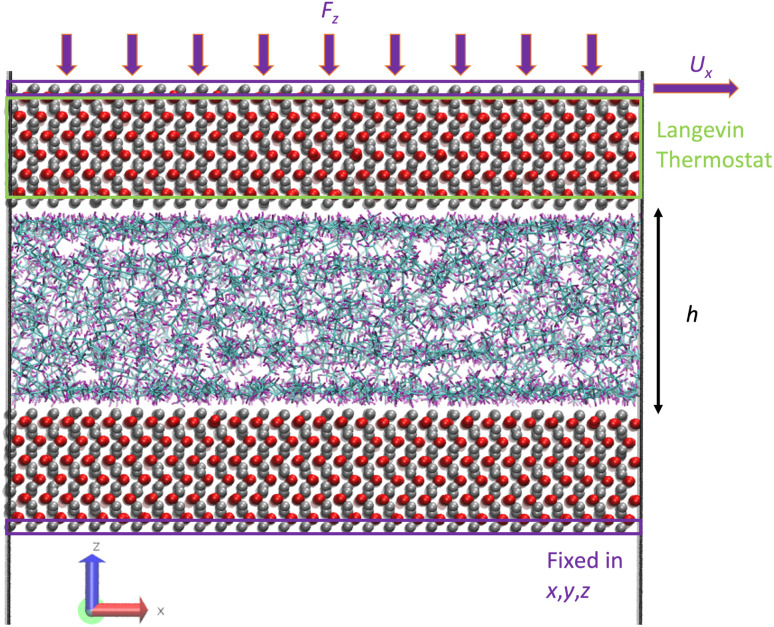
Illustration of the molecular snapshot of system 2 with L-OPLS-AA at 0.1 GPa and 100 °C. The green box represents the thermostating region during the shearing stage of confined NEMD and the two purple boxes represent the outermost iron layers at the top and bottom part of the simulation box. The upper purple box shows the area where the external force *F*_*z*_ is applied, while the purple box at the bottom represents the fixed area of iron atoms. The film thickness of the fluid is equal to *h* and *U*_*x*_ is the external constant velocity which results in an applied shear rate. Carbon atoms are coloured cyan, hydrogen atoms with purple, oxygen atoms with red and iron atoms with silver.

The third step of the simulation included the shearing stage where a shear rate was applied to the system by applying an external velocity at the top outermost layer of iron atoms while continuing to apply an external pressure. The applied shear rate was chosen so that our simulations capture the Newtonian and non-Newtonian (shear thinning) regions. The values were 10^7.5^ and 10^8.5^ s^−1^, respectively. At this stage, the Langevin thermostat was applied at the inner atomic layers of the upper iron oxide slab, as this is known to be a better and more realistic approach to thermostat regions in shearing systems,^23^ instead of applying the thermostat to the fluids, which is known to affect their dynamics.^47^Fig. 7 illustrates the thermostating region during shearing. Again, the outermost bottom layer of the lower iron oxide slab was kept frozen for the whole duration of the simulation. The system was then sheared for 4 ns to ensure a steady state, followed by a production run of 8–80 ns until viscosity converged.

### 4.3 ReaxFF

The ReaxFF protocol was the same as the L-OPLS-AA, with the only difference being the duration of the simulation, as ReaxFF is more computationally expensive than L-OPLS-AA. For this reason, system 3 (case of 450 inserted molecules) was excluded from simulations. The changes were the following.

The simulation timestep was set to 0.25 fs, which has been also used before for investigating the thermal decomposition of phosphate esters on ferrous surfaces with ReaxFF,^42^ while the time constant of the Langevin thermostat was set to 0.01 ps. In addition, the chosen timestep value is included in the suggested timestep range of 0.1–0.5 fs from literature,^48^ which is needed in order to produce reliable dynamics and ensure energy conservation. The heat-quench cycles for simulations at 373 K were performed from 373 K to *T* = 375, 380 and 385 K, respectively. After a 0.15 ns run in the NVT ensemble, the systems were heated during a 0.025 ns run and then they were immediately quenched back to 373 K during another 0.025 ns run in the NVE ensemble. This process occurred two times in order to ensure molecular reorientation.

During the compression step the same pressures as the L-OPLS-AA case were applied, and the compression lasted for 5.8–7.75 ns where the outermost bottom layer of the lower iron oxide slab was kept frozen for the whole duration of the simulation. The simulation ran for long enough until the film thickness fluctuations were lower than 0.07 Å. The film thickness values during the last 0.5 ns were used to determine the average film thickness value needed for the next step of shearing.

During the shearing step the same shear rates as the L-OPLS-AA case were applied, and the system was sheared for 2 ns to ensure a steady state. Again, the film thickness values during the last 0.5 ns were used to determine the average film thickness value needed for the production step of shearing.

Then, the simulation continued with a production run of up to 8 ns, during which viscosity was calculated for each shear rate and trajectory. The simulations at a shear rate of 10^7.5^ s^−1^ were not converged after 8 ns, and due to the computational cost those systems were not run any longer and the ReaxFF results reported are related only to the systems sheared at 10^8.5^ s^−1^.

## Data availability

Data supporting the figures and relevant results within this paper are available upon request on the University of Southampton Institutional Repository, which can be accessed at the following webpage: https://doi.org/10.5258/SOTON/D2436.

## Code availability

The underlying LAMMPS scripts for this study are available upon request on the University of Southampton Institutional Repository, which can be accessed at the following webpage: https://doi.org/10.5258/SOTON/D2436.

## Author contributions

C. S., W. H., M. W., V. B., J. P., C. B., J. F. and P. R. conceived, designed the project and led the collaboration. D. M. designed and carried out the MD simulations, analyzed the results, and wrote the manuscript. D. S. carried out supplementary MD simulations with ReaxFF, analyzed the results and edited the manuscript. All authors contributed to reviewing, revising and editing the manuscript.

## Conflicts of interest

The authors declare no competing interests.

## Supplementary Material

RA-013-D3RA06929J-s001
