# Supplementary material for: Calculating shear viscosity with confined non-equilibrium molecular dynamics: a case study on hematite – PAO-2 lubricant
Source: RSC Adv. 2023 Nov 21;13(48):33994–4002. doi: 10.1039/d3ra06929j (PMC10660148; doi:10.1039/d3ra06929j)
Supplement: RA-013-D3RA06929J-s001 [file RA-013-D3RA06929J-s001.pdf]

Electronic Supplementary Material (ESI) for RSC Advances.
This journal is © The Royal Society of Chemistry 2023

Calculating shear viscosity with confined non-equilibrium molecular dynamics: a case study on hematite - PAO-2 lubricant

Dimitrios Mathas[1], Davide Sarpa[1], Walter Holweger[2], Marcus Wolf[3], Christof Bohnert[4], Vasilios Bakolas[3], Joanna Procelewska[3], Joerg Franke[3], Philipp Rödel[3], and Chris-Kriton Skylaris[1*]

[1]Department of Chemistry, University of Southampton, Highfield, Southampton SO17 1BJ, UK
[2]Mechanical Engineering Department, University of Southampton, Highfield, Southampton SO17 1BJ, UK
[3]Schaeffler Technologies AG & Co. KG, Herzogenaurach, Germany
[4]Department of Mechanical and Process Engineering, RPTU Kaiserslautern-Landau, Gottlieb-Daimler-Str., 67663 Kaiserslautern, Germany
[*]Corresponding Author: Chris-Kriton Skylaris, Department of Chemistry, University of Southampton, Highfield, Southampton SO17 1BJ, UK. Email: C.Skylaris@soton.ac.uk

SUPPLEMENTARY INFORMATION

S.1 Supplementary Figures and Tables

Figure S.1 shows the hexagonal unit cell of α-Fe_2O_3 and Figure S.2 shows the chosen lubricant for this study, 9,10-dimethyloctadecane.

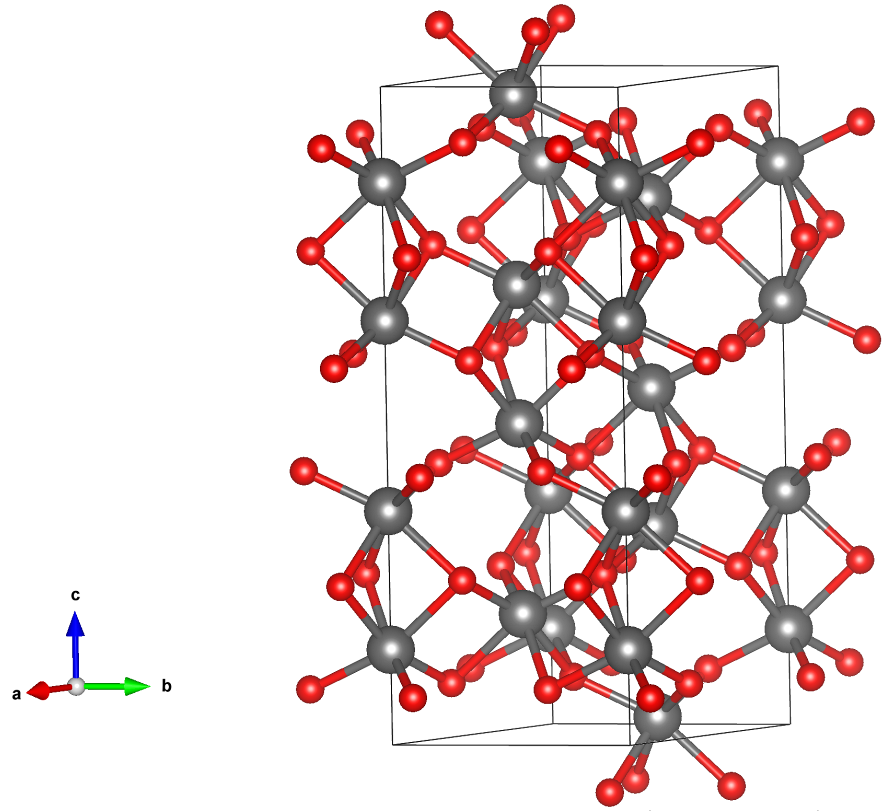

FIGURE S.1: Unit cell of hematite α-Fe_2O_3, a = b = 5.029 Å, c = 13.73 Å, $\alpha = \beta = 90$ °, $\gamma = 120$ °. Iron atoms are coloured with silver and oxygen atoms with red.

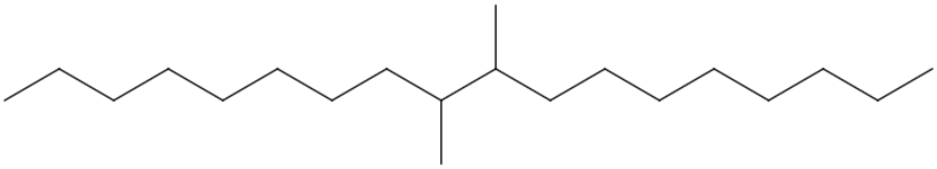

FIGURE S.2: Chemical structure of 9,10-dimethyloctadecane $C_{20}H_{42}$, which has wide industrial applications and its in-depth characterisation at operational conditions is highly desirable.

Figure S.3 shows a molecular snapshot of the system (450 lubricant molecules) after successful compression at a pressure of 0.1 GPa at 100 °C by using the L-OPLS-AA force field.

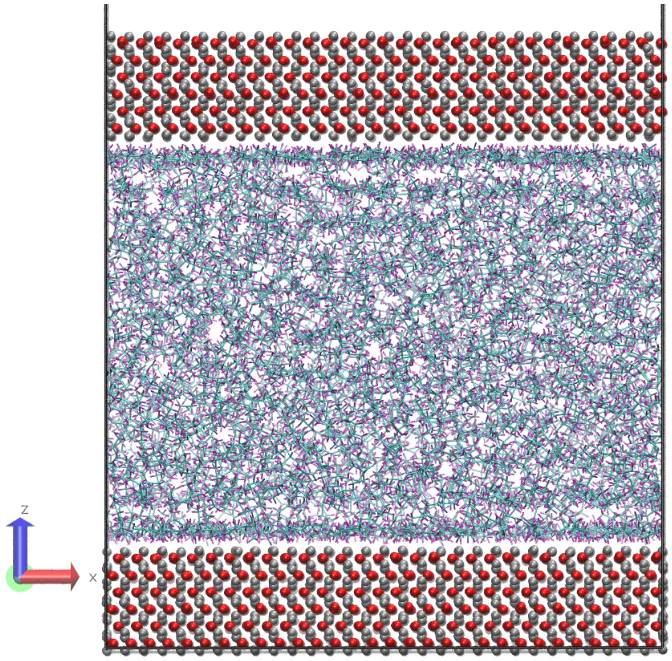

FIGURE S.3: Molecular snapshot of system 3 (450 lubricant molecules), with L-OPLS-AA at 0.1 GPa and 100 °C after the compression stage. Carbon atoms are coloured with cyan, hydrogen atoms with purple, oxygen atoms with red and iron atoms with silver.

Figure S.4 and S.5 show the compression stage during the time evolution of the simulation when using the L-OPLS-AA force field for the remaining two systems (1 and 2).

On the other hand, Figure S.6 and S.7 show the compression stage during the time evolution of the simulation when using the ReaxFF force field for the two different systems (1 and 2). It was found that the ReaxFF force field was slower than the L-OPLS-AA force field in both terms of time required for reaching equilibrium during compression and overall time performance (approximately 44 times slower than L-OPLS-AA).

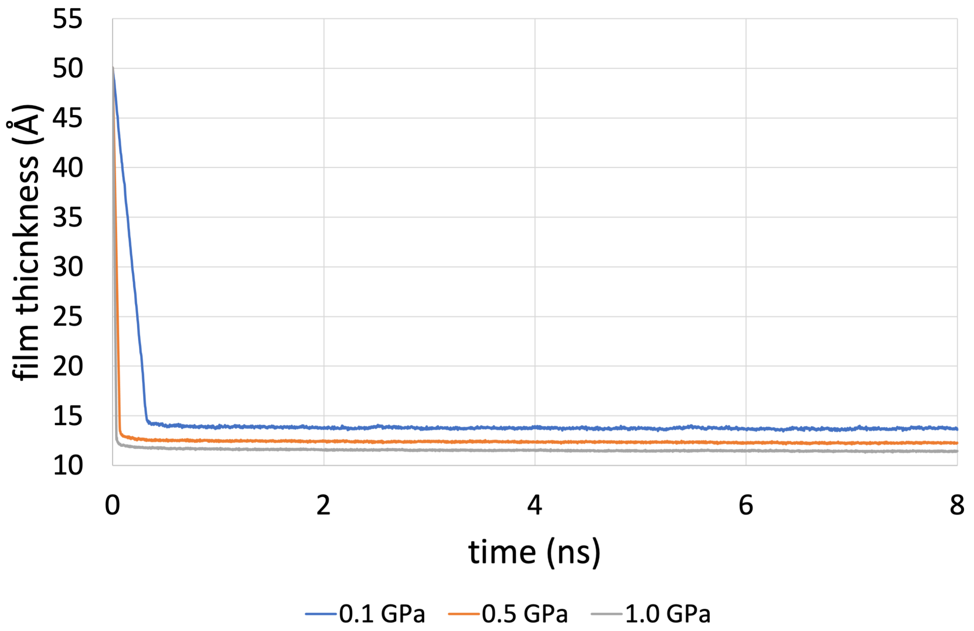

FIGURE S.4: Film thickness of system 1 (100 lubricant molecules), with L-OPLS-AA at 100 °C during the compression stage of 8 ns. For all three pressures, there was a rapid compression and the film thickness oscillated around a constant value.

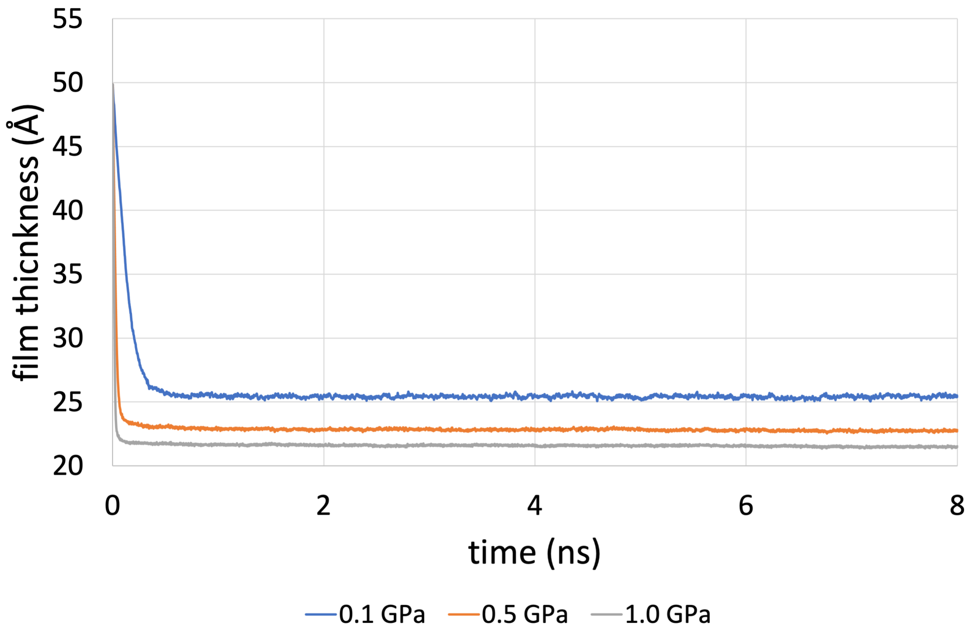

FIGURE S.5: Film thickness of system 2 (200 lubricant molecules), with L-OPLS-AA at 100 °C during the compression stage of 8 ns. For all three pressures, there was a rapid compression and the film thickness oscillated around a constant value.

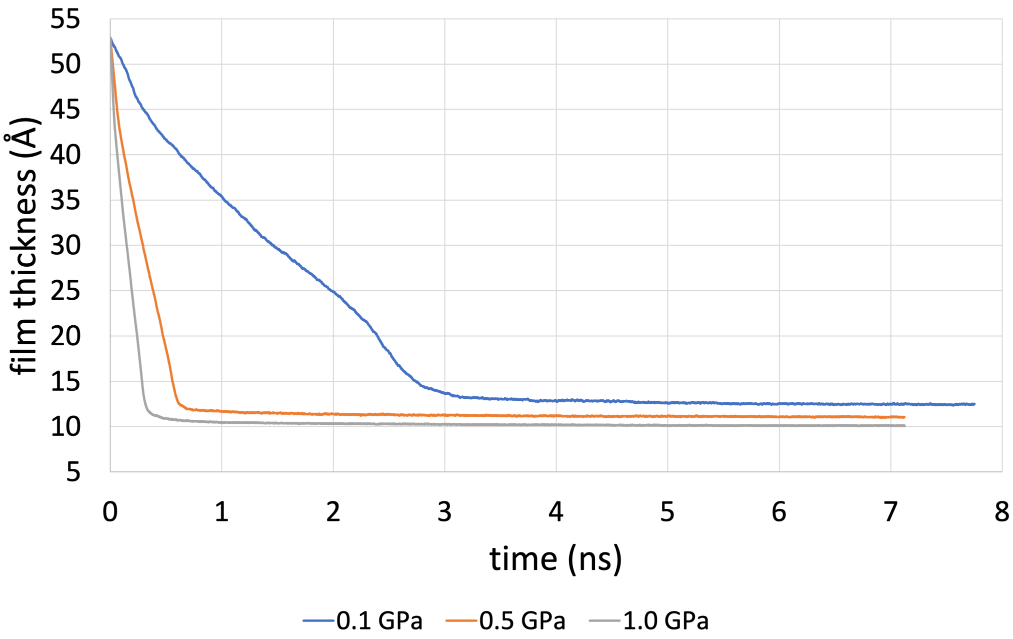

FIGURE S.6: Film thickness of system 1 (100 lubricant molecules), with ReaxFF at 100 °C during the compression stage. As we decrease pressure, the simulation needs to run for longer so that the system reaches a fully-compressed state.

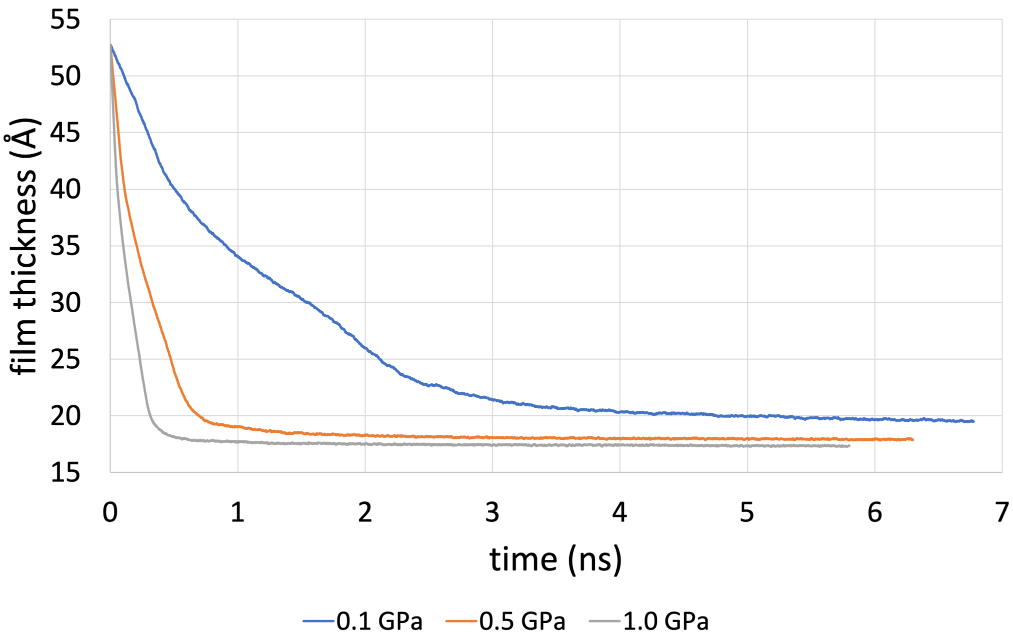

FIGURE S.7: Film thickness of system 2 (200 lubricant molecules), with ReaxFF at 100 °C during the compression stage. Again, as we decrease pressure, the simulation needs to run for longer so that the system reaches a fully-compressed state.

The time-averaged viscosity during the shearing stage of the production run for systems 1, 2 and 3 can be seen in Figures S.8, S.9 and S.10, respectively, for the case of L-OPLS-AA.

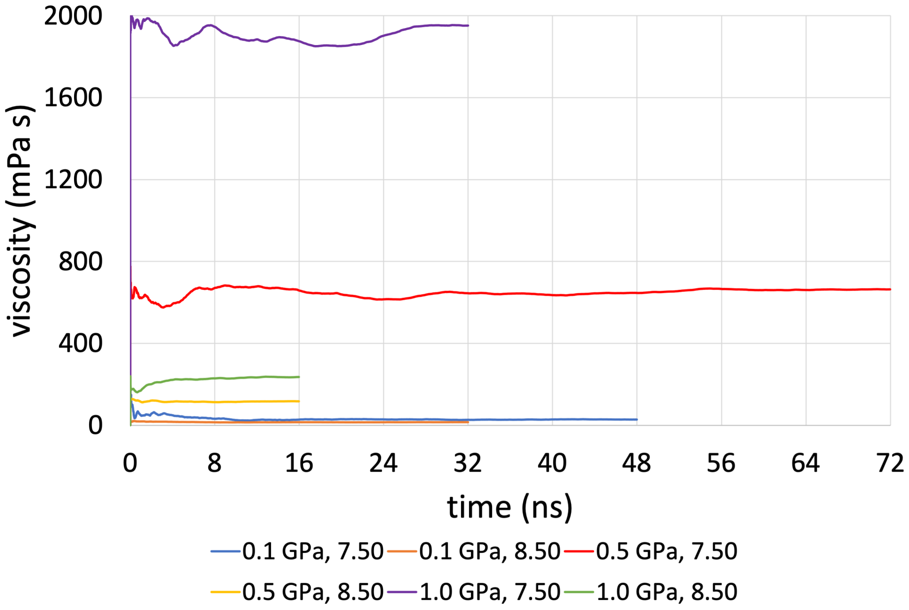

FIGURE S.8: Average viscosity of system 1 (100 lubricant molecules), with L-OPLS-AA at a pressure range from 0.1 to 1.0 GPa at 100 °C and at a shear rate range of $10^{7.5}$ - $10^{8.5}$ s^{-1}.

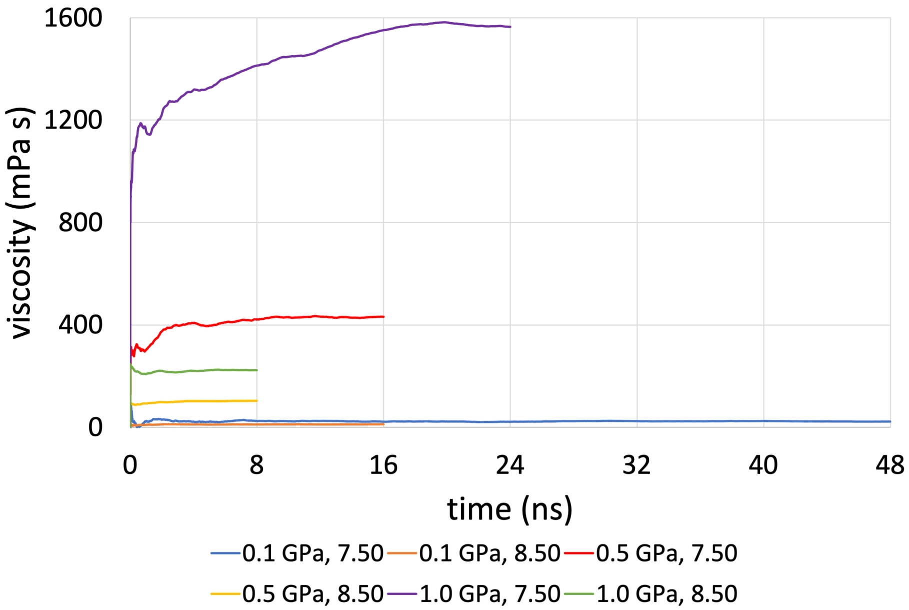

FIGURE S.9: Average viscosity of system 2 (200 lubricant molecules), with L-OPLS-AA at a pressure range from 0.1 to 1.0 GPa at 100 °C and at a shear rate range of $10^{7.5}$ - $10^{8.5}$ s^{-1}.

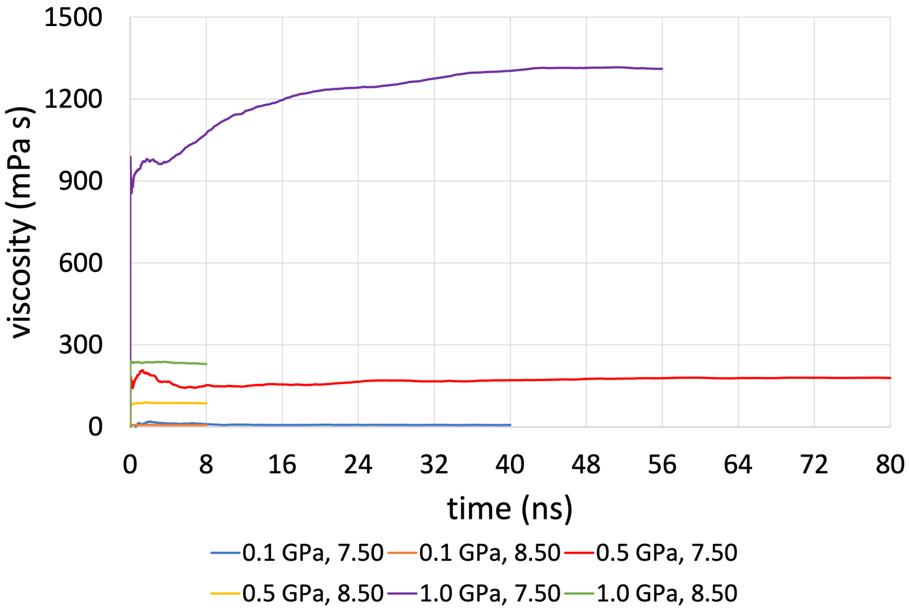

FIGURE S.10: Average viscosity of system 3 (450 lubricant molecules), with L-OPLS-AA at a pressure range from 0.1 to 1.0 GPa at 100 °C and at a shear rate range of $10^{7.5}$ - $10^{8.5}$ s^{-1}.

The following Figures (S.11 - S.15) show the very interesting behaviour of viscosity as a function of the number of lubricant molecules confined within the iron oxide surfaces, which are compared against the bulk NEMD simulations (system 4).

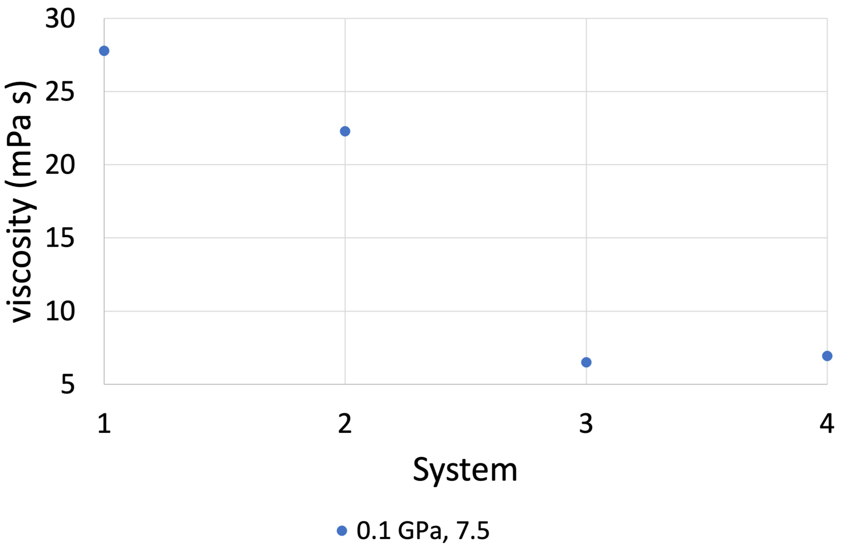

FIGURE S.11: Viscosity results comparison between confined NEMD simulations (system 1, 2 and 3) and bulk NEMD simulations (system 4) at $P = 0.1$ GPa and $\log(\dot{\gamma}[\mathrm{s}^{-1}]) = 7.5$, by using the L-OPLS-AA force field. As we increase the number of confined lubricant molecules we approach bulk behaviour of viscosity. System 1, 2 and 3 contain 100, 200 and 450 lubricant molecules, respectively.

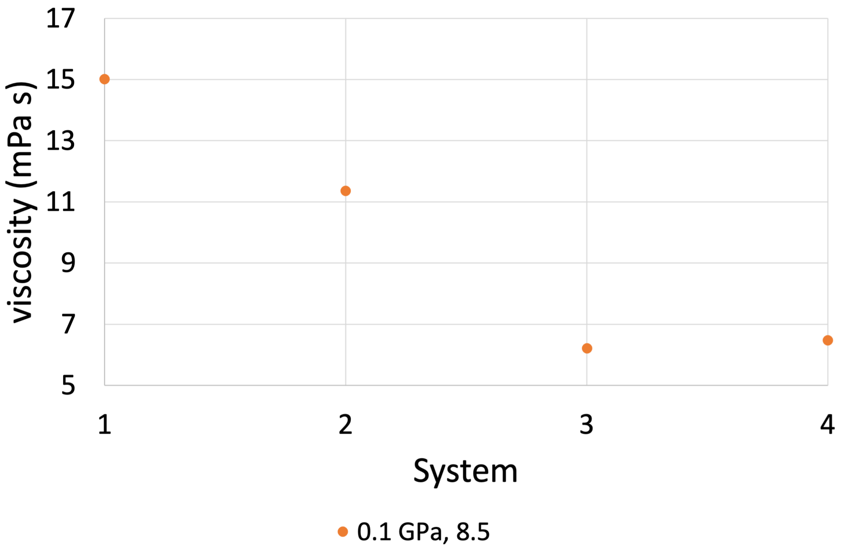

FIGURE S.12: Viscosity results comparison between confined NEMD simulations (system 1, 2 and 3) and bulk NEMD simulations (system 4) at $P = 0.1$ GPa and $\log(\dot{\gamma}[\mathrm{s}^{-1}]) = 8.5$, by using the L-OPLS-AA force field. As we increase the number of confined lubricant molecules we approach bulk behaviour of viscosity. System 1, 2 and 3 contain 100, 200 and 450 lubricant molecules, respectively.

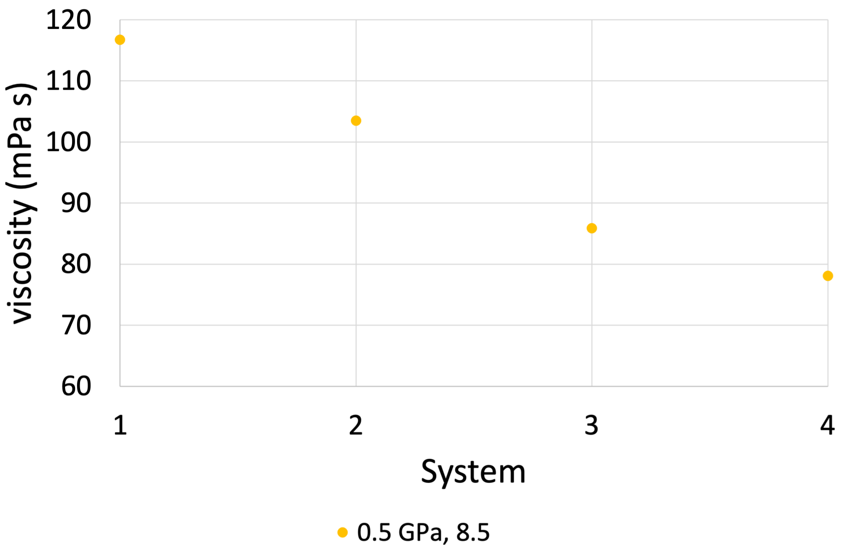

FIGURE S.13: Viscosity results comparison between confined NEMD simulations (system 1, 2 and 3) and bulk NEMD simulations (system 4) at $P = 0.5$ GPa and $\log(\dot{\gamma}[\mathrm{s}^{-1}]) = 8.5$, by using the L-OPLS-AA force field. As we increase the number of confined lubricant molecules we approach bulk behaviour of viscosity. System 1, 2 and 3 contain 100, 200 and 450 lubricant molecules, respectively.

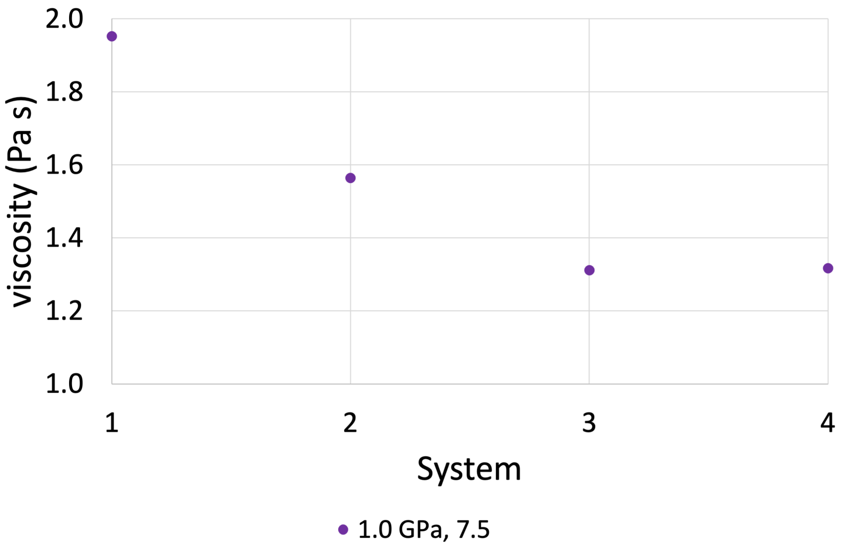

FIGURE S.14: Viscosity results comparison between confined NEMD simulations (system 1, 2 and 3) and bulk NEMD simulations (system 4) at $P = 1.0$ GPa and $\log(\dot{\gamma}[\mathrm{s}^{-1}]) = 7.5$, by using the L-OPLS-AA force field. As we increase the number of confined lubricant molecules we approach bulk behaviour of viscosity. System 1, 2 and 3 contain 100, 200 and 450 lubricant molecules, respectively.

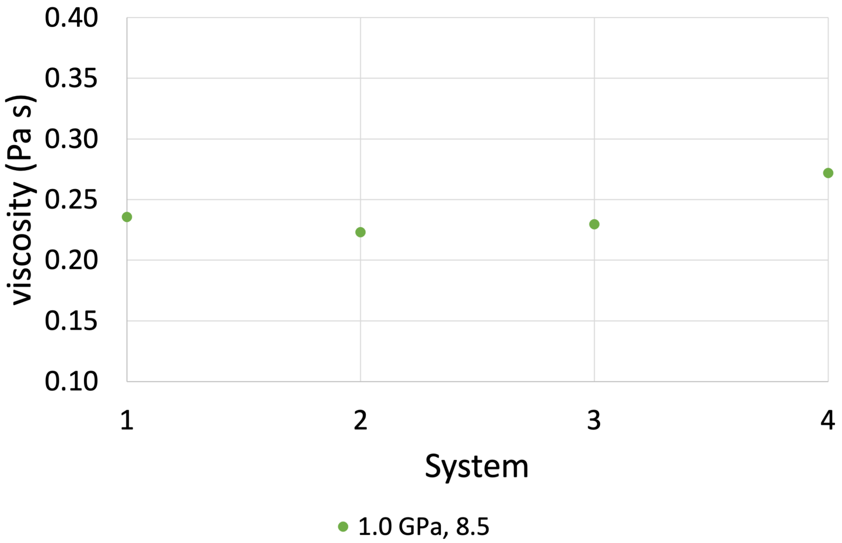

FIGURE S.15: Viscosity results comparison between confined NEMD simulations (system 1, 2 and 3) and bulk NEMD simulations (system 4) at $P = 1.0$ GPa and $\log(\dot{\gamma}[\mathrm{s}^{-1}]) = 8.5$, by using the L-OPLS-AA force field. At this high pressure - high shear rate regime we notice that all confined systems have similar viscosity behaviour with the bulk liquid. System 1, 2 and 3 contain 100, 200 and 450 lubricant molecules, respectively.

Figure S.16 shows the velocity profile of a confined NEMD simulation with L-OPLS-AA for system 3, at a chosen shear rate of $10^{8.5}$ s^{-1} and at a pressure of 1.0 GPa. The simulation box (across z) is divided into equally spaced regions. Then, the velocity vector component (x-dimension) of atoms that exist in each region at a particular timestep is averaged, and then averaged again over different time intervals every 5 ps, to improve statistics during the last 4 ns of the production run. The resulting velocity profile is linear, as described in simple Couette flow. To maintain the same shear rate in simulations at different pressures, the applied velocity at the top edge of the simulation box has to be adjusted accordingly, so as to take into account the change in the film thickness arising from each applied pressure.

Similarly, Figure S.17 shows the velocity profile of a confined NEMD simulation with ReaxFF for system 2, at a chosen shear rate of $10^{8.5}$ s^{-1} and at a pressure of 0.1 GPa. The simulation box (across z) is divided into equally spaced regions. Then, the velocity vector component (x-dimension) of atoms that exist in each region at a particular timestep is averaged, and then averaged again over different time intervals every 1.25 ps, to improve statistics during the 1 ns production run.

The use of the L-OPLS-AA force field resulted in a more linear velocity profile than ReaxFF. This difference can be attributed to the sampling rate of the velocities, which was over a wider interval in the L-OPLS-AA case as it was for 4 ns compared to 1 ns for ReaxFF.

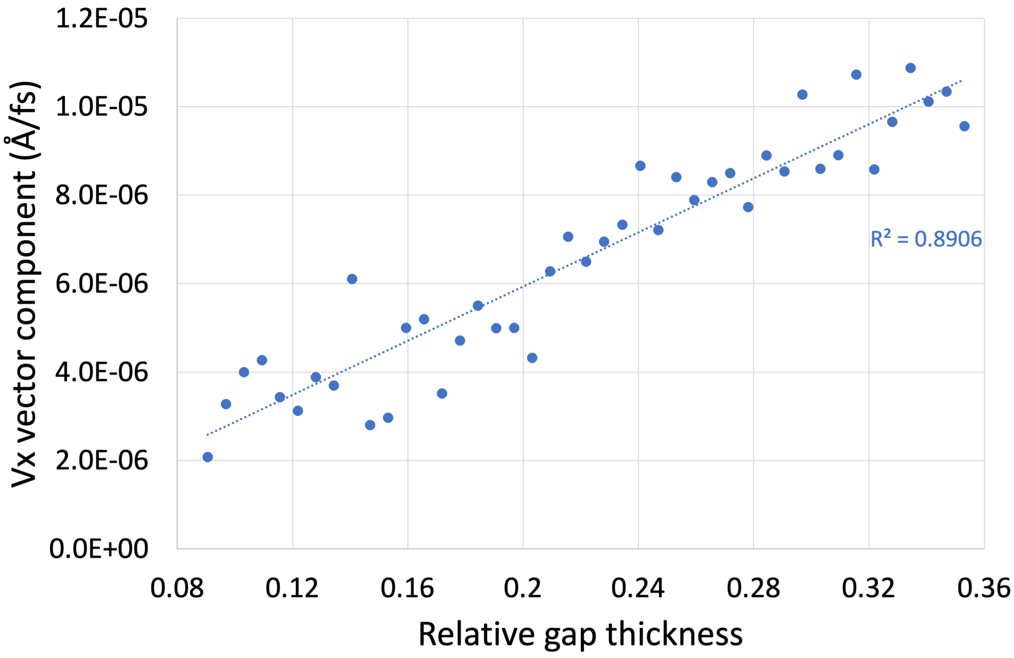

FIGURE S.16: Velocity profile example of 9,10-dimethyloctadecane (450 molecules) at 100 °C, during confined NEMD with L-OPLS-AA at a chosen shear rate of $10^{8.5}$ s^{-1} and at a pressure of 1.0 GPa.

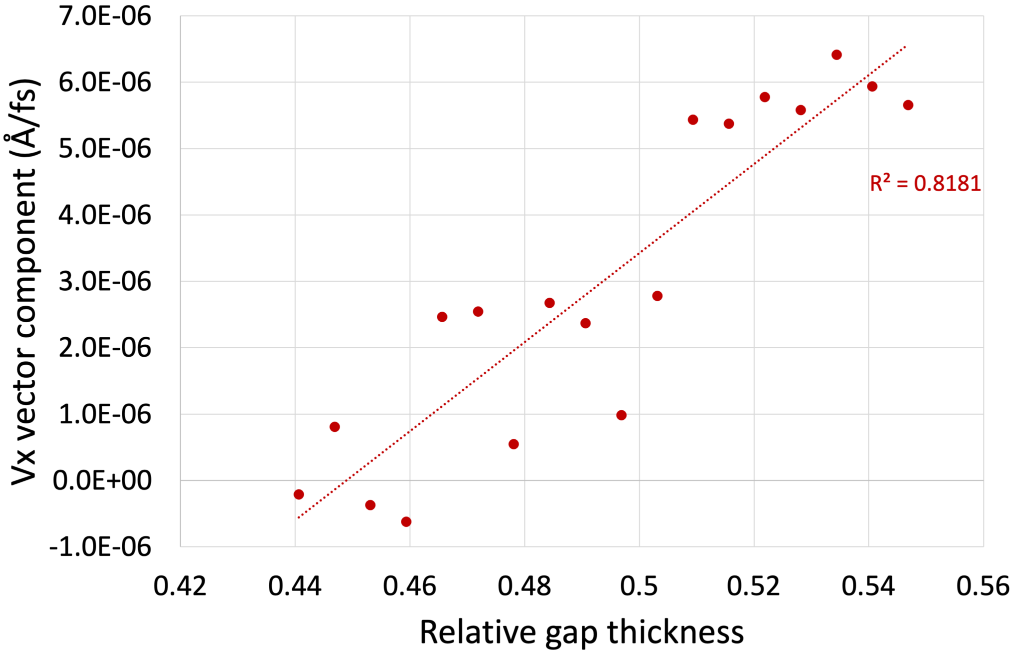

FIGURE S.17: Velocity profile example of 9,10-dimethyloctadecane (200 molecules) at 100 °C, during confined NEMD with ReaxFF at a chosen shear rate of $10^{8.5}$ s^{-1} and at a pressure of 0.1 GPa.

Table S.1 summarises the simulations performed and Table S.2 shows the time duration of the simulation for each system and pressure using the ReaxFF force field.

Table S.1: Simulations performed for confined NEMD. Note that for each case, three independent trajectories were generated.

**Lubricant molecules**	**System**	**log shear rate**	**Force field**	P **(GPa)**
100	1	7.50	L-OPLS-AA	0.1
200	2	7.50	L-OPLS-AA	0.1
450	3	7.50	L-OPLS-AA	0.1
100	1	7.50	ReaxFF	0.1
200	2	7.50	ReaxFF	0.1
100	1	7.50	L-OPLS-AA	0.5
200	2	7.50	L-OPLS-AA	0.5
450	3	7.50	L-OPLS-AA	0.5
100	1	7.50	ReaxFF	0.5
200	2	7.50	ReaxFF	0.5
100	1	7.50	L-OPLS-AA	1.0
200	2	7.50	L-OPLS-AA	1.0
450	3	7.50	L-OPLS-AA	1.0
100	1	7.50	ReaxFF	1.0
200	2	7.50	ReaxFF	1.0
100	1	8.50	L-OPLS-AA	0.1
200	2	8.50	L-OPLS-AA	0.1
450	3	8.50	L-OPLS-AA	0.1
100	1	8.50	ReaxFF	0.1
200	2	8.50	ReaxFF	0.1
100	1	8.50	L-OPLS-AA	0.5
200	2	8.50	L-OPLS-AA	0.5
450	3	8.50	L-OPLS-AA	0.5
100	1	8.50	ReaxFF	0.5
200	2	8.50	ReaxFF	0.5
100	1	8.50	L-OPLS-AA	1.0
200	2	8.50	L-OPLS-AA	1.0
450	3	8.50	L-OPLS-AA	1.0
100	1	8.50	ReaxFF	1.0
200	2	8.50	ReaxFF	1.0

Table S.2: Simulation time for the case of ReaxFF, at a pressure range from 0.1 to 1.0 GPa at 100 °C. For each compression and system, three independent trajectories were used, in order to increase viscosity accuracy.

**Lubricant molecules**	**System**	**Simulation time (ns)**	P **(GPa)**	**Trajectory**
100	1	7.75	0.1	traj_1
100	1	7.75	0.1	traj_2
100	1	7.75	0.1	traj_3
100	1	7.125	0.5	traj_1
100	1	7.125	0.5	traj_2
100	1	7.125	0.5	traj_3
100	1	7.125	1.0	traj_1
100	1	7.125	1.0	traj_2
100	1	7.125	1.0	traj_3
200	2	6.775	0.1	traj_1
200	2	6.775	0.1	traj_2
200	2	6.775	0.1	traj_3
200	2	6.3	0.5	traj_1
200	2	6.3	0.5	traj_2
200	2	6.3	0.5	traj_3
200	2	5.8	1.0	traj_1
200	2	5.8	1.0	traj_2
200	2	5.8	1.0	traj_3
